# Dysregulation of Ceruloplasmin, α2-Macroglobulin, and Alpha-2-HS-Glycoprotein in Transfusion-Dependent Thalassemia

**DOI:** 10.1155/ah/2179600

**Published:** 2025-05-19

**Authors:** Afshan Sumera, Ammu K. Radhakrishnan, Soon Keng Cheong, Abdul Aziz Baba

**Affiliations:** ^1^Department of Pathology, School of Medicine, University of Central Lancashire, Preston, UK; ^2^School of Medicine, International Medical University, Kuala Lumpur, Malaysia; ^3^Jeffrey Cheah School of Medicine and Health Sciences, Monash University Malaysia, Jalan Lagoon Selatan, Sunway 47500, Malaysia; ^4^Universiti Tunku Abdul Rahman, Sungai Long Campus, Kuala Lumpur, Malaysia

**Keywords:** α2-macroglobulin (A2M), alpha-2-HS-glycoprotein (AHSG), ceruloplasmin (CP), transfusion-dependent thalassemia (TDT)

## Abstract

Transfusion-dependent thalassemia (TDT) is a severe inherited anemia characterized by impaired synthesis of hemoglobin chains. Disease progression and TDT severity are potentially linked to oxidative stress and protein damage. This study aimed to explore the expression patterns of ceruloplasmin (CP), α2-macroglobulin (A2M), and alpha-2-HS-glycoprotein (AHSG) in TDT serum through quantitative proteomic profiling. The results were validated using enzyme-linked immunosorbent assays (ELISA). The study participants were divided into three groups based on the duration of blood transfusion. Age and gender-matched normal individuals served as controls. The results revealed the downregulation of these proteins. The reduced levels of these proteins may contribute to tissue damage in TDT patients, primarily due to increased oxidative stress. For example, decreased CP levels can disrupt iron and copper metabolism, leading to heightened oxidative stress and rendering red blood cell membranes more susceptible to rupture due to active oxygen radicals. In summary, CP, A2M, and AHSG association with iron metabolism, inflammation, and oxidative stress underscores their potential relevance in understanding TDT's pathogenesis and progression. These findings may pave the way for improved diagnostic and therapeutic strategies for TDT patients.

## 1. Introduction

Deficiency of beta-globin chains in transfusion-dependent thalassemia (TDT) disrupts the balance of alpha- (α-) and beta- (β-) globin chains in red blood cells [1], causing unpaired alpha-globin chains to form toxic methemoglobin (meth-Hb) [2]. This process releases reactive iron, inducing oxidative stress in the cell membrane and proteins, increasing intracellular calcium levels and apoptosis [1]. TDT symptoms include ineffective erythropoiesis, hemolysis, and chronic anemia. The primary treatment, regular blood transfusions, though necessary for managing anemia, can lead to iron overload, oxidative stress, and tissue damage [3].

Oxidative stress and protein damage are implicated in disease progression and severity [4]. In men experiencing oxidative stress, proteins such as ceruloplasmin (CP), α2-macroglobulin (A2M), and alpha-2-HS-glycoprotein (AHSG) were upregulated in seminal plasma, reflecting stress and metabolic responses [5]. These same proteins are studied as biomarkers in TDT due to their roles in iron metabolism, inflammation, and oxidative stress [6–8]. For example, CP, a copper-binding glycoprotein, facilitates iron transport across cell membranes [9] and protects erythrocyte membranes from oxygen radicals [10, 11]. Reduced CP levels disrupt iron and copper metabolism, increasing oxidative stress and tissue damage [12]. A2M, a protease inhibitor [13], combats misfolded proteins induced by oxidative stress [14]. AHSG, a glycoprotein, is required for biological processes such as endocytosis, brain development, and the formation of bone tissue [15] and has associations with diabetes [16], renal disease [17], and cancer [18]. AHSG deficiency in mice is linked to hepcidin deficiency [8], disrupting iron regulation and leading to iron overload and oxidative stress [19]. In thalassemia, ineffective erythropoiesis suppresses hepcidin, causing spontaneous iron excess and further oxidative stress [20].

Mass spectrometry (MS)-based proteomics is an emerging field that studies the changes in the expression of proteins in various tissues and diseases [21, 22]. Nineteen differentially expressed proteins (DEPs) in liver cells in response to iron overload have been identified in previous studies [23, 24] Molecular and biological pathways analysis showed that most of these dysregulated proteins are involved in processes such as energy metabolism [25], oxidative stress [26], gene expression, and cell cycle regulation [27]. In addition, some of these DEPs were reportedly involved in endocytosis, hypercoagulable state [28], cell injury response, hemolysis [29], antiapoptosis, and apoptotic mitochondrial changes [30].

While the association of recently identified dysregulated proteins with thalassemia is still being investigated, these proteins may contribute to a better understanding of the pathophysiology of TDT in the future [21]. This study aimed to elucidate the differentially expressed CP, A2M, and AHSG proteins in serum from TDT and controls by performing quantitative serum proteomics profiling.

## 2. Materials and Methods

### 2.1. Patients' Selection

A case-control study was conducted with 41 TDT cases and 35 age- and gender-matched controls. All admitted and outpatient-diagnosed TDT patients undergoing TDT treatment between 30th June 2020 and 30th January 2021 at the Tunku Azizah Women and Children Institute of the Kuala Lumpur Hospital, Malaysia, were invited to participate in this study. Written informed consent was taken from all participants recruited for this study. The cases included in this study were stratified into three groups: (i) newly diagnosed cases with a history of blood transfusion < 5 years, (ii) cases with 5–10 years of blood transfusion and (iii) cases with a blood transfusion history of > 10 years. The controls were age and gender-matched normal subjects. The following inclusion and exclusion criteria were used to recruit study subjects.

#### 2.1.1. Inclusion Criteria

a. Thalassemia patients.
  i. Diagnosed cases of TDT.
  ii. No evidence of concurrent infection.
  iii. With or without a recent blood transfusion.
  iv. Age group 1-17 years-old.
b. Healthy controls.
  i. Not a thalassemia carrier.
  ii. No evidence of concurrent infection.
  iii. No history of any malignant disorder.
  iv. Age group 1–17 years old.

#### 2.1.2. Exclusion Criteria

Subjects with evidence of concurrent infection, hospitalized thalassemia patients with complications, and those unwilling to participate were excluded from the study.

### 2.2. Ethics Approval

The study complied with the ethical principles outlined in the Declaration of Helsinki and the Malaysian Good Clinical Practice Guideline. This study was approved by the International Medical University (IMU) Joint Committee on Research and Ethics (IMUJC). Ethical approval was also obtained from the Malaysian Ministry of Health's Medical Research and Ethics Committee (MREC # KKM/NIHSEC/P20-2360(12) dated 09-Dec-2020).

### 2.3. Preparation of Samples

A total of 2 mL of blood was collected from each subject into a 5 mL tube (BD Vacutainer® plastic serum tube). The abundance proteins such as albumin and globulins were removed from the serum using a commercial kit [Pierce™ Albumin/IgG Removal Kit, Thermo Fisher Scientific, USA]. Following this step, the Bradford method [31] was used to quantify proteins in the serum and each protein sample. Following this, according to the kit's protocol, the samples were prepared for the liquid chromatography-MS/MS analysis using the EasyPep™ Mini MS Sample Prep Kit (Thermo Fisher Scientific, USA). Briefly, a volume of serum containing 100 μg of protein was transferred into a clean microcentrifuge tube, and the volume was adjusted to 100 μL using the lysis solution (provided with the kit). This preparation was used for the protein reduction step, where 50 μL of the reduction and alkylation enzymatic solutions (supplied with the kit) were added to the sample. The content was gently mixed after each solution, and the tubes were incubated at 95°C for 10 min to allow reduction and alkylation reactions. Then, the samples were allowed to cool to room temperature before being processed for protein digestion.

For the protein digestion step, 50 μL of Trypsin/Lys-C protease enzyme (provided with the kit) was added to the samples and incubated at 37^o^C for 3 h in a shaking water bath. Then, 50 μL of digestion stop solution (provided with the kit) was added and mixed gently. Each sample was transferred to a peptide clean-up column (supplied with the kit), placed in a clean 1.5 mL tube, and centrifuged (3000 g for 2 min). The flow-through was discarded, and 300 μL of wash solution A (provided with the kit) was added to the column, placed in a 1.5 mL tube, and centrifuged (3000 g for 2 min). The column was placed in a fresh 1.5 mL and 300 μL of wash solution B (provided with the kit) and centrifuged (3000 g for 2 min). The peptide clean-up columns were transferred into fresh microcentrifuge tubes, and 300 μL of elution buffer (provided with the kit) was added to each tube and centrifuged. The column was discarded, and the eluent and the tubes containing the peptides were dried overnight at room temperature using a vacuum centrifuge.

The dried samples were re-suspended in 0.1% FA before label-free quantification using the LC-MS/MS approach (Agilent 6550 Quadrupole Time-of-Flight [QTOF] coupled with Agilent Nanoflow UHPLC and ChipCube). The LC-MS results were further validated using commercial enzyme-linked immunosorbent assay (ELISA) kits to quantify human Fetuin-A (AHSG) (Abcam, UK), human A2M (Abcam, UK), and human CP Assay Kit (Colorimetric) (Abcam, UK).

### 2.4. Statistical Analysis

Comparisons between two groups were performed using the Mann–Whitney test, and multiple comparisons were performed using one-way ANOVA using the Statistical Package for the Social Sciences (SPSS) version 18. The data were expressed as mean ± standard error of the mean (SEM). A *p* value less than 0.05 was considered statistically significant.

## 3. Results

### 3.1. Patient Demographics

The demographic data of cases and controls are shown in Table 1. The age range of cases was between 1 and 17 years (Table 2). All 41 cases were beta-thalassemia major (BTM) and were on daily oral iron chelators, except for two patients on oral and subcutaneous iron chelators. Splenectomy was done only in one patient. The age range of controls was 2–16 years. The results of laboratory and other relevant clinical data of cases are presented in Table 2.

### 3.2. Protein Expression

**Figure 1:**
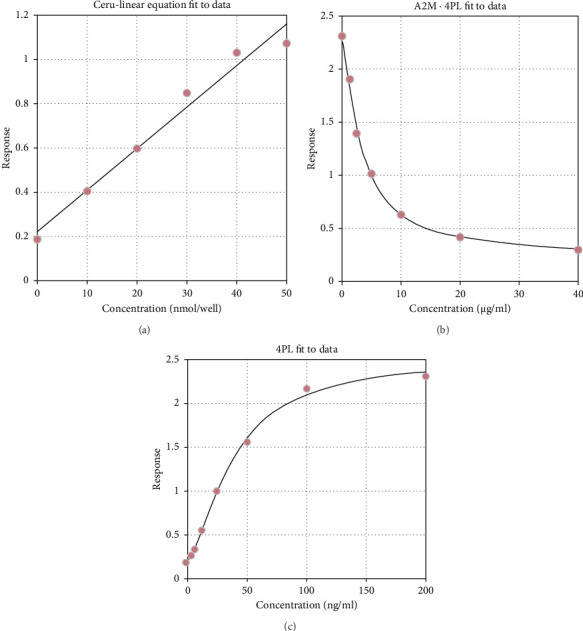
The standard curves (a) ceruloplasmin (b) alpha-2-macroglobulin and (c) alpha-2-HS-glycoprotein (AHSG) obtained using the commercial ELISA.

**Figure 2:**
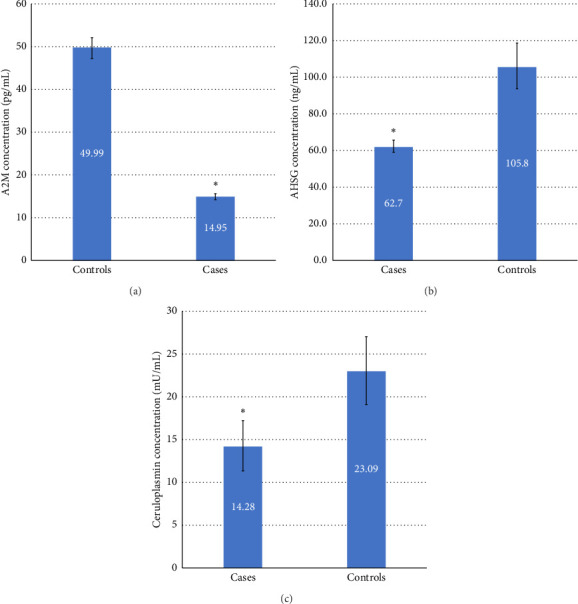
Comparing the mean serum levels of (a) alpha-2-macroglobulin (A2M), (b) alpha-2-HS-glycoprotein (AHSG), and (c) ceruloplasmin (CP) between TDT patients and controls. Data are representative of the standard error of the mean (SEM). (⁣^∗^*p* < 0.05).

The LC-MS/MS analysis showed 51 DEPs between cases and controls. However, only 13 proteins showed statistically significant differential expression (*p* < 0.05) with a fold change greater than 1 (Table 3). The expression of three DEPs, i.e., CP, A2M and AHSG, was validated by ELISA. These DEPs were chosen for validation studies based on their role in oxidative stress and relevance to RBC (Table 3). The ELISA results showed that serum levels of CP in the cases were lower than in the controls (*p* value 0.02) (Table 3). In addition, a significant positive linear association (*R*2 = 0.97) (Figure 1(a)) indicates that the model predicts 97% of the variability in the outcome data, which is a good fit for the data. Similarly, the serum levels of A2M in cases were lower compared to controls (*p* ≤ 0.001) (Figure 2(a)), and there was also a large positive linear association with an *R*2 of 0.998 (Figure 1(b)). The serum levels of AHSG of cases were low compared to controls (*p* ≤ 0.001) (Figure 2(b)), and there was a significant positive linear association with an *R*2 of 0.998 (Figure 1(c)). All three proteins showed differential concentration between cases and controls (Figure 2).

### 3.3. Analysis of Differences Between Groups of Cases

**Figure 3:**
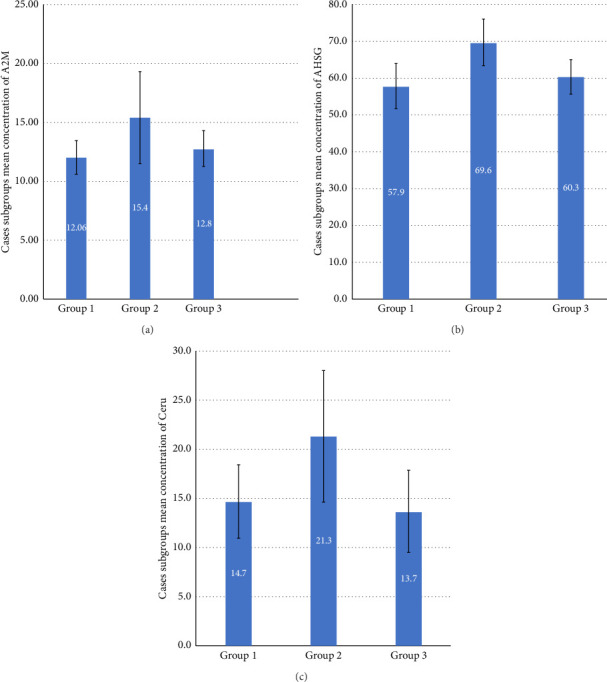
The mean difference in the levels of (a) alpha-2-macroglobulin (A2M); (b) alpha-2-HS-glycoprotein (AHSG); (c) ceruloplasmin (CP) between TDT groups. Group 1 are TDT patients with a history of blood transfusion < 5 years; Group 2 are TDT patients with a history of blood transfusion 5–10 years; Group 3 are TDT patients with a history of blood transfusion > 10 years. Data are representative of the standard error of the mean (SEM).

The mean difference for all three proteins (A2M, AHSG, and CP) among the three groups of TDT patients was also analyzed. Interestingly, within the TDT groups, the serum levels of A2M, AHSG, and CP were higher in group 2 (cases with a history of blood transfusion for 5–10 years) compared to group 1 (blood transfusion < 5 years) and 3 (blood transfusion for > 10 years) (Figure 3).

## 4. Discussion

Iron-mediated organ damage is common in patients with thalassemia syndromes, as dependency on regular blood transfusions leads to oxidative stress. This, in turn, triggers defence mechanisms of cell protection involving numerous proteins [27]. The present study detected significant differential expression of CP, A2M, and AHSG between cases and controls.

A2M is an acute-phase protein and a significant protease inhibitor in the body. It is mainly involved in clearing misfolded/unfolded proteins due to heat or oxidative stress. Researchers studied this protein in sickle cell anemia to explore its role as an acute-phase protein involved during stable-state microvascular occlusions [7]. Another study reported that A2M could be used as a putative biomarker of liver fibrosis to predict the fibrosis stage and minimize the liver biopsy requirement [32]. The A2M levels facilitate inflammatory reactions to inhibit various proteinases and their disposal [33]. In addition, A2M has been implicated in Alzheimer's disease due to its ability to degrade β-amyloid deposition [34]. However, to date, the role of A2M has not been explored in TDT patients. In the present study, A2M was significantly reduced (*p* ≤ 0.001) in the serum from TDT cases compared to controls. Interestingly, further evaluation within cases showed similar levels in groups 1 (blood transfusion < 5 years) and 3 (blood transfusion > 10 years) compared to group 2 (blood transfusion 5–10 years) amongst the TDT cases. The erythropoietic drive and iron homeostasis differ between newly diagnosed TDT cases and chronic multi-transfused patients [35]. A recent study shows a higher level of ineffective erythropoiesis with high serum ferritin and transferrin saturation levels in newly diagnosed thalassemia patients [35]. The explanation could be that increased iron absorption is due to massive erythropoietic activity. The levels of GDF15 are increased in thalassemia [36], produced by erythroid progenitors, resulting in a decreased hepcidin secretion from the liver [35, 36]. The hepcidin suppression of iron absorption takes time; hence, the effect is delayed in newly diagnosed TDT patients (group 1). Therefore, later iron absorption is suppressed by hepcidin, resulting in slightly lower levels of ferritin than in group 2. However, chronic repeated blood transfusions again increase the iron levels in group 3. The downregulation of A2M may also be responsible for this effect, which needs further exploration. Hence, we propose that low levels of A2M can contribute to tissue damage in TDT cases due to oxidative stress.

AHSG (fetuin-A), a blood glycoprotein synthesized in the liver, is mainly involved in brain and bone development. Its role has been studied in cardiovascular disease [37], but its role in thalassemia still needs to be explored. For instance, the downregulation of AHSG in TDT patients may provide new insights into iron overload associated with oxidative stress in TDT cases. AHSG-deficient mice were reported to develop hepcidin deficiency [8]. In this study, AHSG levels were statistically significantly lower (*p* < 0.05) in cases compared to controls. The group analysis results were consistent with A2M results, which show higher levels in group 2 than in groups 1 and 3. Therefore, it can be attributed to higher iron levels in newly diagnosed and chronic TDT cases.

In TDT, chronic transfusions and resulting iron overload can lead to a dysregulation of CP function. Studies have shown that CP levels are often decreased in TDT patients, which may contribute to the development of complications such as iron overload and oxidative stress [6, 38]. CP is a plasma protein involved in iron hemostasis [39] and an antioxidant protein [40], mainly engaged in copper transport, coagulation pathways, and angiogenesis [41]. It is reported that reduced CP levels lead to abnormal iron and copper metabolism that increases oxidative stress, which makes the RBC membrane more vulnerable to rupture due to active oxygen radicals [6]. In 2013, researchers found that the inverse association of CP with ferritin was observed in healthy populations; less CP leads to higher ferritin levels [42]. In the present study, we report the downregulation of CP in TDT patients, which was confirmed by an independent laboratory test (ELISA). The results from the ELISA showed that serum CP concentration was low in TDT compared to controls (*p* < 0.05) (Figure 2). The TDT group analysis showed results comparable with A2M and AHSG group results, i.e., higher levels in group 2 patients than in group 1 and group 3. We propose the same explanation for this difference as we have mentioned for the A2M and AHSG group results. To our knowledge, this is the first report showing significant associations between these proteins and TDT patients.

In conclusion, CP, A2M, and AHSG have emerged as significant prognostic biomarkers in TDT. Their differential expression patterns and decreased serum concentrations observed in TDT patients compared to healthy controls could potentially facilitate the prediction of tissue damage secondary to oxidative stress in these individuals. In addition, their involvement in iron metabolism, inflammation, and oxidative stress underscores their potential relevance in understanding the pathogenesis and progression of TDT. Integrating these biomarkers into clinical practice may provide valuable insights into disease monitoring, treatment optimization, and developing novel therapeutic approaches.

### 4.1. Limitations of the Study

Given the relatively limited patient cohort, confirming the prognostic significance of these proteins necessitates validation in larger, independent populations. Further investigation is warranted to evaluate the clinical applicability of these plasma biomarkers in monitoring thalassemia severity and guiding transfusion decisions. Functional studies examining the relationship between these proteins and cumulative transfusion rates are also imperative. Moreover, future research should explore the identification of critical thresholds for each biomarker relevant to patients with TDT to understand disease severity better.

## Figures and Tables

**Table 1:** Demographics of cases and controls.

			**Frequency**	**Percent (%)**

Cases (*n* = 41)	Race	Malay	38	93
		Chinese	3	7
		Indian	0	0
	Gender	Female	18	44
		Male	23	56

Controls (*n* = 35)	Race	Malay	32	91
		Chinese	2	6
		Indian	1	3
	Gender	Female	12	34
		Male	23	66

**Table 2:** Laboratory data of TDT cases.

Lab parameters	TDT cases group 1	TDT cases group 2	TDT cases group 3
	Transfusion < 5 years	Transfusion 5–10 years	Transfusion > 10 years
	Mean (SEM)	Mean (SEM)	Mean (SEM)
WBC (10^9^/L)	8.8 (±0.7)	7.5 (±0.6)	8.2 (±1.6)
Hb (g/dL)	10.2 (±0.3)	10.6 (±0.3)	10.8 (±0.4)
Platelet (10^9^/L)	406.5 (±21.9)	367.7 (±31.3)	318.1 (±46.7)
Hct (%)	29.4 (±0.8)	31.3 (±1.2)	31.8 (±1.3)
RBC (10^12^/L)	3.7 (±0.1)	4.0 (±0.2)	4.2 (±0.2)
MCV (fL)	78.8 (±0.8)	76.4 (±1.5)	76.7 (±0.9)
MCH (pg)	27.3 (±0.3)	25.6 (±0.6)	26.0 (±0.5)
MCHC (g/dL)	34.6 (±0.2)	33.2 (±0.5)	33.9 (±0.4)
RDW-SD (fL)	43.9 (±1.1)	44.9 (±2.4)	44.6 (±7.4)
Albumin (g/L)	41.2 (±0.5)	42.2 (±0.8)	42.5 (±1.1)
Alkaline Phosphatase (ALP) (U/L)	266.7 (±26.9)	332.3 (±31.4)	185.9 (±37.0)
Alanine transaminase (ALT) (U/L)	59.5 (±19.5)	16.9 (±3.6)	28.5 (±5.3)
Bilirubin, total (Umol/L)	23.6 (±3.4)	37.6 (±6.3)	36.9 (±4.9)
Sodium (nmol/L)	136.7 (±0.3)	137.0 (±0.4)	136.3 (±1.1)
Potassium (nmol/L)	3.9 (±0.1)	3.7 (±0.1)	3.8 (±0.1)
Chloride (nmol/L)	106.8 (±0.5)	106.0 (±0.9)	104.6 (±1.1)
Urea (nmol/L)	4.2 (±0.3)	4.4 (±0.2)	4.0 (±0.5)
Creatinine (Umol/L)	34.4 (±2.7)	42.8 (±3.5)	50.0 (±2.4)
Serum ferritin levels (µg/L)	2066.5 (±230.9)	1484.9 (±535.9)	3960.4 (±910)

*Note:* M/F, male/female; Hb, hemoglobin; Hct, hematocrit.

Abbreviations: MCH, mean corpuscular Hb; MCHC, mean corpuscular Hb concentration; MCV, mean corpuscular volume; RBC, red blood cells; SEM, standard error of mean; WBC, white blood cells.

**Table 3:** Serum proteins with differential expression in controls versus cases.

	Accession number	Frequency (%)		Avg. mass of protein	Description of proteins	Gene name	*p* value	Expression
		Cases	Controls					
1	P00450	100	100	122,205	Ceruloplasmin	*CP*	**0.02**	↓
2	P01019	100	90	53,154	Angiotensinogen	*AGT*	**0.01**	↓
3	P01023	100	100	163,290	Alpha-2-macroglobulin	*A2M*	**0.001**	↓
4	P01024	95	80	187,147	Complement C3	*C3*	**0.02**	↑
5	P02647	100	90	30,778	Apolipoprotein A-I	*APOA1*	**0.01**	↓
6	P02763	100	100	23,512	Alpha-1-acid glycoprotein 1	*ORM1*	**0.02**	↓
7	P02765	100	100	39,341	Alpha-2-HS-glycoprotein	*AHSG*	**0.03**	↓
8	P02774	100	90	52,918	Vitamin D-binding protein	*GC*	**0.02**	↓
9	P02790	25	90	51,676	Hemopexin	*HPX*	**0.001**	↓
10	P04217	100	100	54,254	Alpha-1B-glycoprotein	*A1BG*	**0.03**	↓
11	P06727	100	90	45,372	Apolipoprotein A-IV	*APOA4*	**0.02**	↓
12	P19652	100	100	23,603	Alpha-1-acid glycoprotein 2	*ORM2*	**0.01**	↓
13	P25311	100	90	34,259	Zinc-alpha-2-glycoprotein	*AZGP1*	**0.07**	↑

*Note:* ↓: Downregulated; ↑: Upregulated. Bold values indicate that the differential expression observed are statistically significant (*p* < 0.05).

## Data Availability

Data are available on request from the authors.
